# Electric readout of magnetic stripes in insulators

**DOI:** 10.1038/s41598-019-55565-1

**Published:** 2019-12-13

**Authors:** Yao Chen, Yuki Shiomi, Zhiyong Qiu, Tomohiko Niizeki, Maki Umeda, Eiji Saitoh

**Affiliations:** 1 Institute for Materials Research, Tohoku University, Sendai, 980-8577 Japan; 2 Department of Basic Science, The University of Tokyo, Meguro, Tokyo 153-8902 Japan; 3 Advanced Institute for Materials Research, Tohoku University, Sendai, 980-8577 Japan; 4 Advanced Science Research Center, Japan Atomic Energy Agency, Tokai, 319-1195 Japan; 5 Department of Applied Physics, University of Tokyo, Hongo, Tokyo 113-8656 Japan; 6 Present Address: Key Laboratory of Materials Modification by Laser, Ion, and Electron Beams (Ministry of Education), School of Materials Science and Engineering, Dalian University of Technology, Dalian, 116024 China

**Keywords:** Superconducting properties and materials, Magnetic properties and materials

## Abstract

In superconductors, a topological configuration of the superconducting order parameter called a superconducting vortex carries magnetization. Such a magnetic topological object behaves like a minute particle generating a magnetic flux. Since the flux is localized with a nanometer scale, the vortex provides a nano-scale probe for local magnetic fields. Here we show that information of magnetic stripes in insulators can be read out by using vortices in an adjacent superconductor film as a probe. The orientation and width of magnetic micro stripes are both transcribed into resistance change of the superconductor through the modulation of vortex mobility affected by local magnetization. By changing the direction of external magnetic fields, zero-field resistance changes continuously according to the stripe orientation, and its modulation magnitude reaches up to 100%. The width of the stripes can also be estimated from the oscillatory magnetoresistance. Our results demonstrate a new possibility for non-volatile analog memory devices based on topological objects.

## Introduction

While digital computing devices have dominated electronics and information technology for several decades, analog memory devices may sow the seeds of neuromorphic computing technology. Magnetic structures in antiferromagnetic materials have attracted great attention for potential application to analog memories. Recently, a machine learning system was found to be boosted by using an analog memory effect in antiferromagnet/ferromagnet bilayer devices^1^. By controling internal magnetic structures of the antiferromagnetic layer continuously, they achieved memristive operation of the devices. In the antiferromagnet, the complicated magnetic structures exhibit almost zero net magnetization, which makes them robust against external magnetic disturbance and thus suitable for information storage. However, manipulating and reading magnetic information in antiferromagnets is still an unestablished job, since the spacial scale of such magnetic structures is as small as atomic scale.

In ferro- and ferri-magnetic films with perpendicular magnetic anisotropy, stripe magnetic domain structures reminiscent of antiferromagnets often appear at zero field in a nano- to micrometer-scale, where neighboring domains are arranged antiparallel along the out-of-plane direction. The stripe structures are stabilized via the competition between shape magnetic anisotropy and perpendicular magnetocrystalline anisotropy^2^, when the thickness of magnetic films exceeds a critical thickness^3^. It is known that the orientation of the stripes can be set along an external magnetic field direction to reduce the total anisotropy energy when an in-plane magnetic field greater than the saturation field is applied and then it is withdrawn; the stripe patterns are reconfigurable^2^. The orientation of the stripes can carry information, being a candidate for a principle of analog memories owing to its nonvolatility and robustness against magnetic fluctuations. However, no method for electrical readout of information from magnetic stripes has been found yet.

How can we detect the magnetic stripe information electrically in insulating magnets? Insulator magnets exhibit very week dissipation of magnetic dynamics, advantageous to memories and record medium. In this study, we show a mechanism for electric detection of magnetic-stripe information by using topological objects, superconducting vortices in superconductor/ferrimagnetic-insulator bilayers.

**Figure 1:**
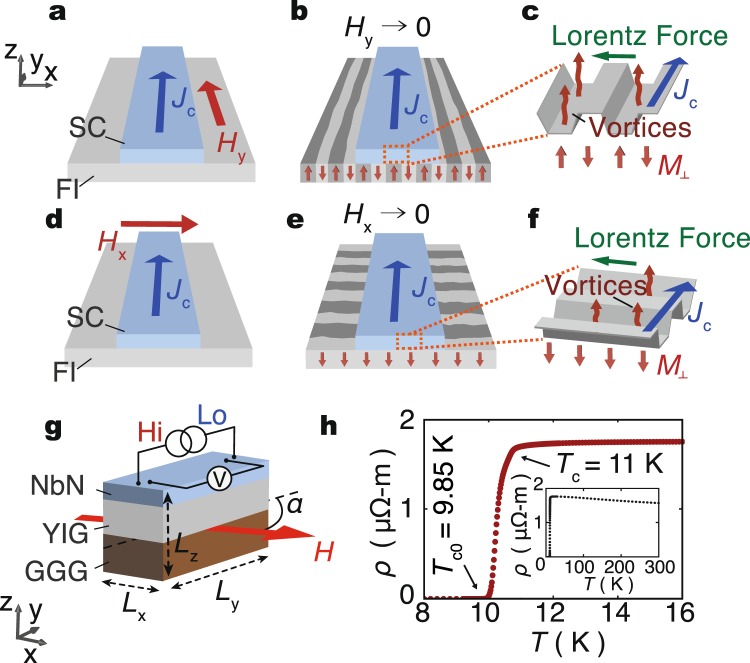
Experimental concept. (**a**) Consider a superconductor (SC)/ferrimagnetic insulator (FI) bilayer sample. By applying an in-plane external magnetic field, *H*, parallel to the *y*-axis and larger than the magnetization saturation field of the ferrimagnetic insulator (FI), the magnetization of FI (YIG) aligns in the same direction as the field, and magnetic domains disappear. (**b**) By withdrawing *H* from (**a**), perpendicularly magnetized stripes along the initially applied field direction appear in the FI layer. (**c**) A close-up view of (**b**). Superconducting vortices are created above the stripes which illustrated as confining potential for vortices. Lorentz force, *F*, acting on the vortices is perpendicular to the bias current. (**d**–**f**) Stripe and vortex configurations when *H* is applied parallel to the *x*-axis. (**g**) Measurement setup of the present study. The sample is a YIG/NbN bilayer film. An in-plane magnetic field, *H*, is applied at a relative angle, *α*, to the current direction. (**h**) Temperature (*T*) dependence of *ρ* for the YIG/NbN sample at *H* = 0. The thermodynamic superconducting transition temperature and the zero resistance temperature are denoted as *T*_c_, and *T*_c0_, respectively. The inset shows the *T* dependence of *ρ* up to 300 K.

A superconducting vortex refers to a quantized magnetic flux in type-II superconductors. In superconducting films put on a magnet, stray magnetic fields created from the magnet give rise to vortices in the superconducting layer^4–7^ and affects the dynamics of vortices. Since the size of the vortex core is less than a micrometer, the magnetostatic interaction of the vortices^8^ allows us to probe micro-size magnetic structures in the magnet. Furthermore, electric detection of the internal magnetic structure in the magnet is possible by measuring resistance caused by vortex motion^9^ in the superconductor film; by applying a bias current to the superconducting layer, the current induces vortex motion along the direction perpendicular to the current, generating voltage parallel to the current via the vortex-Josephson effect, and thus finite resistance is produced: the vortex flow resistance^9^. The flow resistance is proportional to the velocity of the vortex; therefore it provides an electrical method to evaluate the mobility of vortices. When the direction of the vortex motion is perpendicular to the magnetic-stripe direction, magnetic potential barrier at the domain boundaries in the magnet lowers the mobility of the vortices in the superconductor film, giving rise to anisotropy in the vortex flow resistance, illustrated in Fig. 1a–f. By using the anisotropic transport of vortices, information of magnetic stripes can be detected, as demonstrated below.

## Results

Figure 1 schematically illustrates the samples used in the present study and our experimental concepts. The samples are bilayer films comprising a single-crystaline (111) ferrimagnetic insulator Y_3_Fe_5_O_12_ (YIG) layer and a type-II superconductor NbN layer, fabricated on Gd_3_Ga_5_O_12_ (111) substrates (see Methods). Resistivity, *ρ*, of the NbN layer is measured by a 4-wire sensing method (see Fig. 1g). Temperature dependence of *ρ* under zero magnetic field *H* = 0 is shown in Fig. 1h. As the temperature *T* decreases from 300 K, *ρ* slightly increases and then exhibits superconducting transition at *T*_c_ = 11 K. *ρ* sharply drops at *T*_c_ and becomes lower than the measurement limit below *T*_c0_ = 9.85 K.

**Figure 2:**
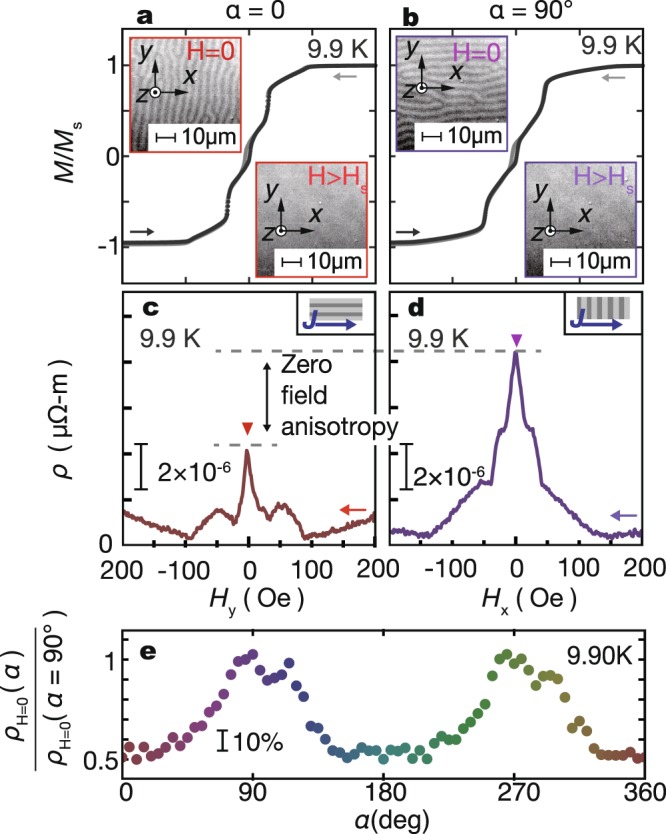
Detection of magnetic stripes via magnetoresistance (MR). (**a**,**b**) Magnetic-field dependence of magnetization of the YIG/NbN sample at *T* = 9.9 K for *α* = 0 and *α* = 90° (for the definition of *α*, see Fig. 1g). We also show the magneto-optic images of the YIG sample at *H* > *H*_s_ and at *H* = 0. Magnetic stripes are observed at *H* = 0. (**c**,**d**) MR measured at 9.9 K, with the inplane *H* applied along the *α* = 0 and *α* = 90° directions. The arrows in (**a**–**d**) denote the field sweep directions. (**e**) The zero-field *ρ* obtained from the MR measurements with different *α* (*ρ*_H=0_(*α*)) values at 9.9 K. The value of *ρ*_H=0_(*α*) is normalized by *ρ*_H=0_(*α* = 90°).

In Fig. 2a,b, we show the magnetization *M* as a function of the in-plane *H* for $H\Vert \hat{y}$ (along the long axis of the sample, see Fig. 1g) and $H\Vert \hat{x}$ (along the short axis) at 9.9 K, together with magneto-optical images of the YIG layer. The magnetization is saturated above around *H*_s_ = 100 Oe. At *H* > *H*_s_, no stripe patterns are recognized in the magneto-optical images (see Fig. 2a,b). In contrast, when *H* is decreased to zero, stripe patterns parallel to the initial *H* direction are clearly seen in the magneto-optical images for the YIG sample. The result shows that an external magnetic field can control the orientation of magnetic stripes.

Figure 2c,d show magnetic field dependence of the resistivity *ρ* at *T* = 9.9 K, slightly higher than *T*_c0_, at which vortices are set to move under a bias current^10^ (see Supplementary Fig. S1), at *α* = 0 and *α* = 90°. Here, *α* is defined as the angle between *H* and the *y*-axis, as shown in Fig. 1g. Importantly, *ρ* exhibits remarkable *α* dependence; as highlighted by triangles in Fig. 2c,d, the zero-*H* resistivity *ρ*(*H* = 0) of the NbN layer shows different values between *α* = 0 and *α* = 90°. The value of *ρ*_α=0_(*H* = 0) is much less than that of *ρ*_*α*=90°_(*H* = 0), in spite of the fact that the temperature and the bias current are same for both setups. As *H* decreases from high fields to zero, stripe domains are created around *H* = 0 in the YIG layer in response to the initial magnetic-field directions, as shown in Fig. 2a,b. Then, vortices created in the NbN layer by the stray field from the stripe domains in the YIG layer cause anisotroic vortex flow resistance in the NbN layer. The greater resistivity in the configuration where the direction of the current is perpendicular to that of the magnetic stripes is consistent with the aforementioned stronger potential barrier effect for vortices at domain boundaries. The dependence of *ρ* on *α* remains even after we insert an insulating 10 nm SiO_2_ film between the YIG and NbN (see Supplementary Fig. S3).

**Figure 3:**
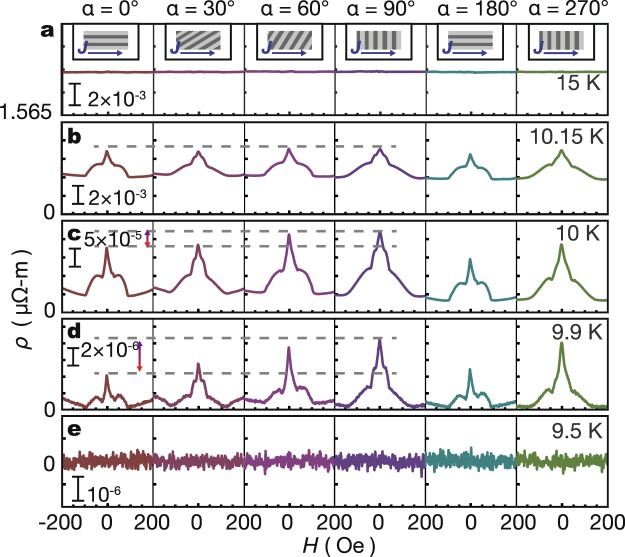
*T* dependence of magnetoresistance. (**a**–**e**) MR measured at *T* = 15 K, 10.5 K, 10 K, 9.9 K and 9.5 K with various *α* values. Field sweep direction is from positive to negative value.

The *α* dependence of *ρ*_*α*_(*H* = 0), obtained from the magnetoresistance (MR) measurements, at 9.9 K is shown in Fig. 2e. When the *H* direction *α* is increased from zero, *ρ*_*α*_(*H* = 0) shows a maximum at *α* = 90° and then decreases to show a minimum at *α* = 180°. By further increasing *α*, *ρ*_*α*_(*H* = 0) again shows a maximum at 270° and a minimum at 360°. The 180° periodicity can be attributed to the stripe symmetry of the magnetic stripes in the YIG. It is notable that the resistance ratio *ρ*_*α*_(*H* = 0)/*ρ*_*α*=90°_(*H* = 0) changes from 50% to 100% upon changing *α*, which is much greater than the magnitude of the conventional anisotropic magnetoresistance (AMR) in magnets. The result shows that the orientation of magnetic stripes can be detected electrically using vortex dynamics in the vicinity of *T*_c0_, in which vortices are set to move. In Fig. 3a–e, we show MR at various *T* and *α* values. We observe no *α* dependence in the normal state of NbN (*T* = 15 K) and the zero resistivity state (*T* = 9.5 K). We also notice that, by increasing *T*, the zero field anisotropy [*ρ*_H=0_(*α* = 90°) − *ρ*_H=0_(*α* = 0)]/*ρ*_H=0_(*α* = 90°) decreases. The temperature dependence of the anisotropic transport can be understood by the competition between the pinning force, the driving force and the thermal fluctuation force^11^. However, it requires more detailed measurements to evaluate the strength of pinning. Such measurements are beyond the scope of this study. One intuitive interpretation of the temperature dependence is at a higher temperature, vortices are thermally activated and the anisotropic pinning force from magnetic stripes become less dominate in the transport^11,12^. The systematic *T* and *α* dependence of *ρ*_*α*_(*H* = 0) are shown in Supplementary Fig. S4.

**Figure 4:**
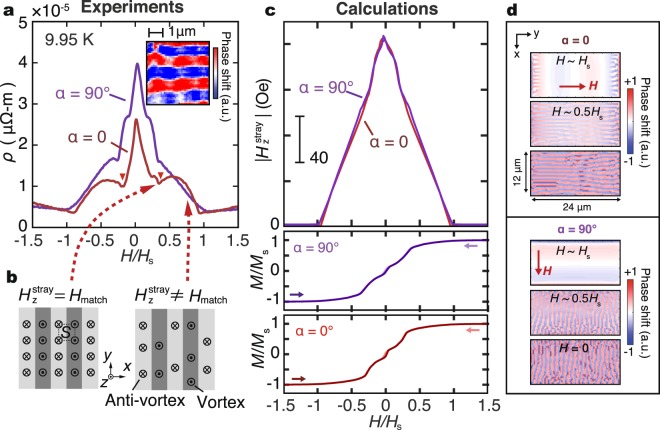
Comparison of magnetoresistance and calculation. (**a**) Magnetoresistance (MR) measured at 9.95 K with *H* applied in the *α* = 0 and *α* = 90° directions. *H* is normalized by the saturation field *H*_s_ of the YIG layer for each *α*. The inset shows an MFM image at the same *T* for the YIG sample with *H* = 0. (**b**) A schematic illustration of the vortex matching effect. *S* is the cross sectional area facing *H*. (**c**) Calculated results of the magnetization process in the YIG and the magnitude of the perpendicular stray field |*H*_*z*_^stray^| of the YIG sample. (**d**) Calculated MFM images at different magnetic fields.

Let us turn to the change of *ρ* with *H*. Figure 4a compares the MR curves measured for *α* = 0 and *α* = 90° at 9.95 K, where *H* is normalized by *H*_s_. At a high *H* range (|*H*| > *H*_s_ = 100 Oe for *α* = 0) where magnetic stripes are absent, *ρ* monotonically increases with increasing *H* both for *α* = 0 and *α* = 90°. This trivial *H* dependence is due to the suppression of superconductivity due to the strong magnetic fields. In the *H* range where magnetic stripes are present (|*H*| < *H*_s_), we found that *ρ*_*α*=90°_(*H*) is greater than *ρ*_*α*=0_(*H*), indicating that the magnetic stripes affect the vortex transport properties even in the presence of an external magnetic field. *ρ* in both setups reaches a minimum around *H*_s_, where magnetic stripes disappear and the YIG layer becomes a single domain magnetic state.

In the range of −*H*_s_ < *H* < *H*_s_, *ρ*_*α*=0_(*H*) surprisingly shows oscillatory behavior: dips (local minima) at *H* = |0.35*H*_s_| appear, as highlighted by the red triangles in Fig. 4a. The oscillation can be explained by a vortex matching effect in periodic pinning potential; when the period of the vortex lattice is commensurate with the period of pinning potential, the vortex motion is slowed down, resulting in electric resistance decrease^13–16^, (see Fig. 4b). For the *α* = 90° case, in contrast, *ρ* monotonically decreases as *H* increases towards *H*_s_, where several small structures are observed. The matching effect is not remarkable in *ρ*_*α*=90°_(*H*) because the driving force is parallel to the pinning channel.

A numerical calculation also supports the above scenario. We carried out numerical calculations on the magnetization process of the YIG layer under in-plane magnetic fields to estimate the *H* dependence of the *z* component of the stray field (see Method for detials). As shown in Fig. 4c, the calculated magnetization curve of the YIG layer reproduces the *M*-*H* curves experimentally observed in our YIG/NbN sample (see Fig. 2a,b); a hysteresis loop at very low fields and a sudden jump of *M* around *H* = *H*_s_/2 are clearly reproduced in our calculations. Magnetic stripes are also reproduced: based on results from the micromagnetic simulation, we calculated MFM images at different magnetic fields. The results are shown in Fig. 4d. Note that the external field can manipulate the direction of magnetic stripes. The width of magnetic stripes is about 600 nm, slightly narrower than the actual value(~1 μm). This mismatch between simulation and experiment may come from the different thickness-to-length ratios between the model and the real sample. This may also cause a tiny difference in *H*_s_ between calculations and experiments, but it does not affect the conclusion of our discussion.

The calculated averaged magnitude of the perpendicular stray field (|*H*_z_^stray^|) at 20 nm above the YIG surface as a function of *H* is shown in Fig. 4c. The calculated stray fields for both *α* = 90° and 0 decrease monotonically with |*H*|. No significant difference appears between *α* = 90° and 0. The calculation result confirms that the origin of the anisotropic resistivity dose not lie in the stray fields but in the anisotropic mobilities of vortices. The vortex matching effect appears to take place when the period of the vortex lattice matches the width of magnetic stripes^4,16^. From the calculated stray field, the |*H*_z_^stray^| value at the matching condition is estimated to be *H* = 80 Oe. In previous studies^4,13–16^, the matching condition was estimated as Φ_0_/*S*, where Φ_0_ is the magnetic flux quantum and *S* is the cross sectional area facing *H*. In the present study, *S* = *d*^2^ (where *d* stands for the domain width. See Fig. 4b) and the matching magnetic field *H* is *μ*_0_*H* = Φ_0_/*d*^2^. We thereby estimated the width of magnetic stripes as *d* = 510 nm. This magnitude almost agrees with the width (~1 μm, see the inset to Fig. 4a) measured by magnetic force microscopy (MFM) at 10 K.

**Figure 5:**
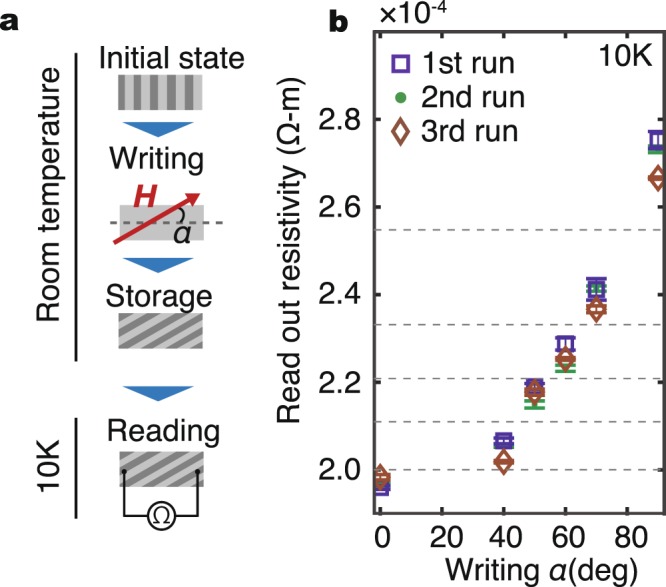
Writing and reading of magnetic stripes. (**a**) Procedures for the writing and reading. First, an in-plane magnetic field at an angle *α* to the *y*-axis is applied and then it is withdrawn. This procedure writes stripes with the angle *α* in the YIG. Then, we drop the temperature down to 10 K and measure the resistance of the NbN layer. (**b**) Results of three runs of writing and reading. *α* indicates the stripe directions. The error bars represent the standard deviation of measurements. Dotted lines are guides for the eye.

Harnessing the vortex anisotropic transports, we demonstrated writing and reading operation of magnetic stripes, as shown in Fig. 5. The measurement sequence is depicted in Fig. 5a. First, a weak in-plane magnetic field *H* = 200 Oe (>*H*_s_) with an in-plane angle, *α*, is applied to the sample, followed by withdrawing the field to create stripes in the YIG layer at room temperature. This is the writing process. Then we decrease the temperature down to 10 K and measure the resistance of the NbN layer at zero field; the orientation of magnetic stripes does not change by cooling because of the nonvolatile nature of the stripes. This sequence is repeated several times at different *α* values to check the reproducibility of the device operation. Figure 5b shows results of three runs. In each run, we performed the writing-reading operation at *α* = 0, 40°, 50°, 60°, 70°, and 90°. For all the *α* values, the device exhibits a good reproducibility for three runs of writing and reading analog information. The magnitude of the resistance change at *α* = 0 and *α* = 90° is as large as 40%, which can differentiate the direction of magnetic stripes reliably. The present data demonstrates that the orientation of magnetic stripes can be distinguished in terms of the vortex transport with good reproducibility.

## Discussion

We have demonstrated the electric readout of magnetic stripes in an insulator YIG using the vortex dynamics in superconductors. The stray field from magnetic stripes in YIG introduces superconducting vortices in an adjacent NbN layer, and the orientation and width of magnetic stripes were detected in terms of vortex flow resistance. Significant anisotropy (50–100%) in the vortex flow resistance was observed, which enables the efficient electric detection of orientation of magnetic stripes. Repetitive operation of the writing and reading processe has been confirmed to show the validity of our concept.

Anisotropic transport phenomenon in superconductors induced by fixed nanofabricated artificial grooves^11^, magnetic lines^12^, or so-called domain wall superocnductivity was reported in ferromagnet/superconductor hybrids^17–19^. In the nanostructured grooves and magnetic lines, they act as fixed guidance of vortices, different from our study that demonstrates writing and reading operations from rewritable and tunable magnetic stripes in insulator films. In the domain wall superconductivity, the stray field is higher than the upper critical field (*H*_c2_) of the superconducting layer; the superconductivity remains only at the region above domain walls, since the stray field is smaller than that at the interior of the stripe regions. The localization of superconducting order parameter above domain walls was studied theoretically by Aladyshkin *et al*.^20^ and observed experimentally later^21–23^.

The YIG layer exhibits smaller remnant in-plane magnetization (~0.1 *M*_s_) and perpendicular magnetic anisotropy. This ensures significant amount of out-of-plane local magnetization at zero field. Therefore the spontaneous nucleation of vortices^6,7^ in the NbN layer is realized, and it enables us to readout the analog information via zero-field resistivity. From the magnetic field-temperature diagram (see Supplemental Fig. S1), we obtained $-{\mu }_{0}\frac{d{H}_{{\rm{c2}}}}{dT}{|}_{{T}_{{\rm{c}}}}=3.91\,{\rm{T}}/{\rm{K}}$, which yields to *μ*_0_*H*_c2_(*T* = 0) ≈ 29.7 T and a coherence length *ξ*(*T* = 0) ≈ 3.3 nm^24^. In the dirty limit, we estimate the penetration length as *λ*(*T* = 0) ≈ 436 nm^25^. The corresponding values of *μ*_0_*H*_c2_, *ξ* and *λ* at 9.95 K are respectively 4.6 T, 10.5 nm and 723 nm. Using the criterion from Iavarone *etal*.^7^, vortices will spontaneous nuclear once the perpendicular component of the magnetization is larger than 5.6 A/m, which is much smaller than the simulation amount (~10000 A/m) at zero field.

Furthermore, a stray field from our YIG layer is so small that it does not exceed *H*_c2_ of the NbN layer; the transport anisotropy in the present study is due mainly to different vortex mobilities in different stripe directions. This allows us to use magnetic materials with less magnetization and anisotropy than the previous works^18,26^, enabling us to observe the vortex matching effect in the MR curve. The interaction between superconducting vortex and magnetic layer were reported in many aspects, such as the spontaneous nucleation of vortices^6,7^, the pinning of magnetic domains by superconducting vortex^27^, the guided vortex motion detected by magneto-optical imaging^28^ and transport measurements^26^. However, this work first demonstrates the magnet/superconductor hybrids can serve as nonvolatile analog storage elements. We point out the same mechanism may apply to other topological objects. For example, topological objects in magnets, known as skyrmions, also couple with currents and stray fields from magnetic stripes and can be used to readout stripe information under room temperature.

## Methods

### Sample fabrication

An epitaxial Y_3_Fe_5_O_12_ (111) film of the thickness 6-μm was grown on a Gd_3_Ga_5_O_12_ (111) 0.5-mm-thick substrate by the liquid phase epitaxy^29^. The sample was cut into small pieces with 1 × 3 mm^2^ rectangular surface area ((*L*_x_, *L*_y_, *L*_z_) = (1 mm, 3 mm, 0.5 mm), see Fig. 1g). Then, NbN films with the thickness of 20 nm were sputtered at room temperature on the YIG films. For the NbN sputtering, a Nb target was sputtered in a N_2_-Ar mixture gas^30^. The YIG(111) film exhibits out-of-plane magnetic anisotropy and magnetic stripe patterns below the magnetization saturation field.

### Resistivity and magnetization measurement

Resistivity (*ρ*) of the YIG/NbN samples was measured by a 4-wire sensing method in a physical property measurement system (PPMS; Quantum Design, Inc.). The sample was fixed on a PPMS rotator option chip with GE varnish and attached to a rotator rod. The longitudinal voltage was measured with a nanovoltmeter (2182A; Keithley, Inc.) when the bias current with the density ~10^8^ A/m^2^ was applied to the sample with an a.c. current source (6221; Keithley, Inc.). The delta mode method was used in the *ρ* measurements to reduce noise levels. The MR was measured by sweeping magnetic fields along a direction at an angle (*α*) to the *y*-axis. Magnetization measurements were also performed in PPMS by using a vibrating sample magnetometer (VSM) option. Domain structures in a bare YIG film were observed by polarized light microscopy at room temperature and also by magnetic force microscopy (MFM) at *T* = 10 K.

### Micromagnetic calculation

The micromagnetic calculation was performed using OOMMF^31^, based on the Landau-Lifshitz-Gilbert equation:

1 $$\frac{d{\boldsymbol{m}}}{dt}=-\gamma {\boldsymbol{m}}\times {{\boldsymbol{H}}}_{{\rm{eff}}}+\alpha {\boldsymbol{m}}\times \frac{d{\boldsymbol{m}}}{dt}$$

where *γ*, ***m*** and *α* are the gyromagnetic ratio, unit vector of the magnetization, and the Gilbert damping coefficient, respectively. The effective field ***H***_eff_ includes the external field, the exchange field, the demagnetization field, and the cubic anisotropy field. The sample size used in the simulation along the *x*, *y*, *z* directions are 12 μm, 24 μm and 2 μm, respectively, and the sample was divided into unit cells of 100 × 100 × 100 nm^3^.

## Supplementary information

Supplementary Information

## Data Availability

The data used to support the findings of this study are available from the corresponding author upon request.
