# Supplementary material for: Electric readout of magnetic stripes in insulators
Source: Sci Rep. 2019 Dec 13;9:19052. doi: 10.1038/s41598-019-55565-1 (PMC6911107; doi:10.1038/s41598-019-55565-1)
Supplement: Supplementary file 1 — Supplementary Information [file 41598_2019_55565_MOESM1_ESM.pdf]

Supplementary Information of "Electric readout of magnetic stripes in insulators"

Yao Chen*[1], Yuki Shiomi[2], Zhiyong Qiu[1,6], Tomohiko Niizeki[3], Maki Umeda[1] & Eiji Saitoh[1,3,4,5]

[1]Institute for Materials Research, Tohoku University, Sendai 980-8577, Japan

[2]Department of Basic Science, The University of Tokyo, Meguro, Tokyo 153-8902, Japan

[3]Advanced Institute for Materials Research, Tohoku University, Sendai 980-8577, Japan

[4]Advanced Science Research Center, Japan Atomic Energy Agency, Tokai 319-1195, Japan

[5]Department of Applied Physics, University of Tokyo, Hongo, Tokyo 113-8656, Japan

[6](Present address) Key Laboratory of Materials Modification by Laser, Ion, and Electron Beams (Ministry of Education), School of Materials Science and Engineering, Dalian University of Technology, Dalian 116024, China

*Correspondence to y.chen@imr.tohoku.ac.jp

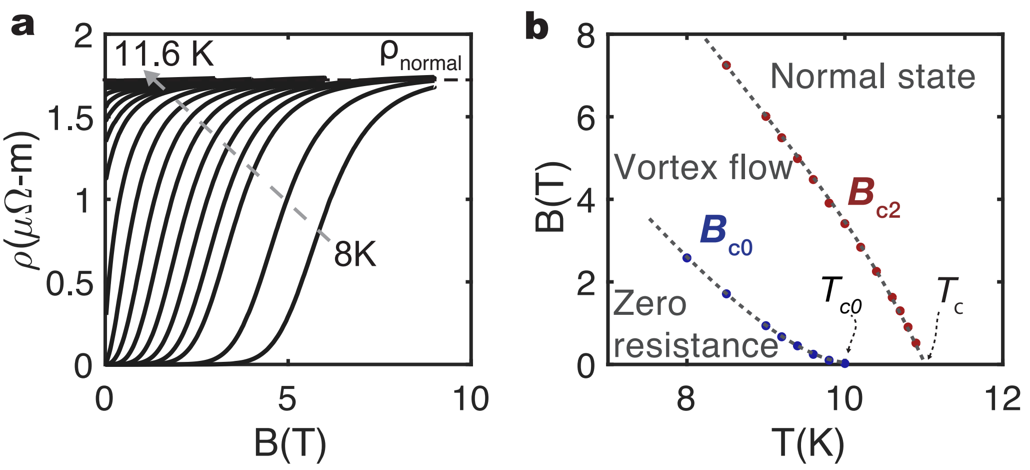

Figure S1: Upper critical fields determined from the MR results. (a) Magnetic field dependence of the resistivity (ρ) measured in the temperature regime from 8 K to 11.6 K. The normal state resistivity is denoted by ρ_{normal}. (b) Temperature dependence of the zero-resistance-field B_{c0} and the upper critical field B_{c2} of the NbN film determined from the data in (a). Here, B_{c0} and B_{c2} are defined by $\rho(B_{\mathrm{c0}}) = 10^{-4}\rho_{\mathrm{normal}}$ and $\rho(B_{\mathrm{c2}}) = 0.95\rho_{\mathrm{normal}}$, respectively. T_{c} and T_{c0} stand for the critical temperatures; T_{c} is the onset temperature of superconductivity, while T_{c0} the temperature where the resistivity becomes zero. T_{c} and T_{c0} are obtained by extrapolating B_{c2} and B_{c0} data to the x-axis, respectively. Dotted curves are guides for eyes. In the vortex flow region, unconventional magnetoresistance is observed, as discussed in the main text.

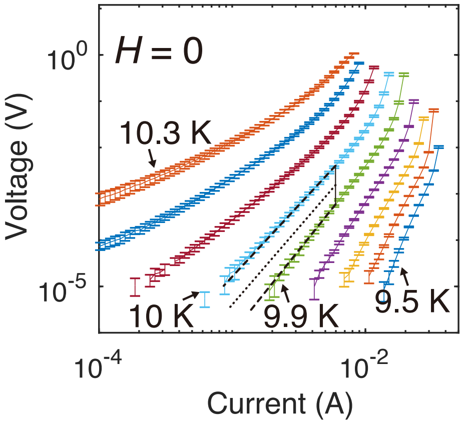

Figure S2: Current-Voltage ($I - V$) curves for YIG/NbN film. Results are measured in the temperature range from 9.5 K to 10.3 K, at $H = 0$. At the same bias current, the low current parts of the $I - V$ curves have opposite concavities for $T < 9.9$ K and $T > 10.0$ K, suggesting a vortex solid-liquid phase transition occurs near 9.95 K[1]. The resistance vanishes in the limit of $I \to 0$ for $T < 9.9$ K, which indicates the vortex solid phase. For $T > 10.0$ K, finite resistance remains at $I \to 0$, which indicates the vortex liquid phase. Superconducting $B - T$ phase diagram similar to Fig. S1b can be extracted from systematic measurements of the $I - V$ curves at various magnetic fields. The error bars represent the standard deviation of measurements. Dashed lines are tangents to $I - V$ curve at the same bias current. The dotted line is a guide for the eye.

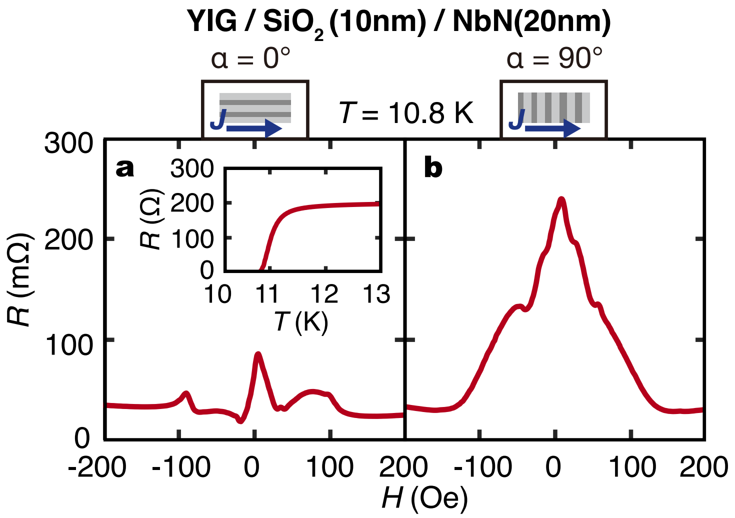

Figure S3: Magnetoresistance (MR) in a YIG/SiO_2 (10nm)/NbN sample. (a),(b) MR measured at 10.8 K, with the inplane H applied along the $\alpha = 0$ and 90° directions, respectively. The inset in (a) shows the temperature dependence of resistance at $H = 0$. The resistance magnitude at the zero magnetic field is different between $\alpha = 0$ and 90° even when an insulating layer is inserted between YIG and NbN layers. This result clearly indicates that the magnetostatic interaction is responsible for the large anisotropy in the zero-field resistance.

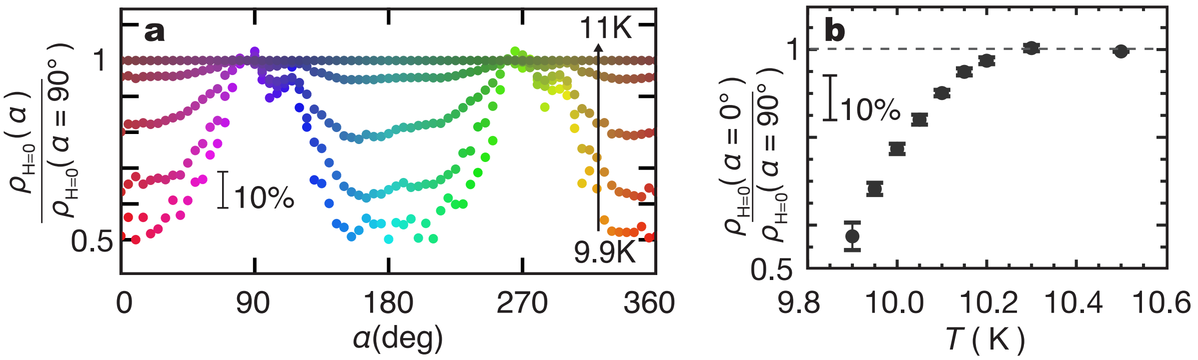

Figure S4: Dependence of the zero-field resistivity on temperature and in-plane field angle. (a) The zero-field resistivity ($\rho_{\mathrm{H}=0}(\alpha)$) obtained from the MR measurements with different α values at 9.9 K, 9.95 K, 10.05 K, 10.2 K and 11 K. With increasing T, the anisotropy in the zero field resistivity decreases for all α values. (b) T dependence of the anisotropy in the zero field resistivity $\rho_{\mathrm{H}=0}(\alpha = 0)/\rho_{\mathrm{H}=0}(\alpha = 90°)$.

1. Strachan, D. R. *et al.* Do Superconductors Have Zero Resistance in a Magnetic Field? *Physical Review Letters* **87**, 067007 (2001).
